# Characterization of Intestinal Microbiomes of Hirschsprung’s Disease Patients with or without Enterocolitis Using Illumina-MiSeq High-Throughput Sequencing

**DOI:** 10.1371/journal.pone.0162079

**Published:** 2016-09-07

**Authors:** Yuqing Li, Valeriy Poroyko, Zhilong Yan, Liya Pan, Yi Feng, Peihua Zhao, Zhoulonglong Xie, Li Hong

**Affiliations:** 1 Department of Clinical Nutrition, Shanghai Children’s Medical Center, Shanghai Jiaotong University School of Medicine, Shanghai, China; 2 Department of Medical Oncology &Therapeutics Research, City of Hope, Duarte, California, United States of America; 3 Department of Surgery, Shanghai Children’s Medical Center, Shanghai Jiaotong University School of Medicine, Shanghai, China; 4 Institute of Molecular Ecology and Evolution, SKLEC & IECR, East China Normal University, Shanghai, China; AC Camargo Cancer Hospital, BRAZIL

## Abstract

Hirschsprung-associated enterocolitis (HAEC) is a life-threatening complication of Hirschsprung’s disease (HD). Although the pathological mechanisms are still unclear, studies have shown that HAEC has a close relationship with the disturbance of intestinal microbiota. This study aimed to investigate the characteristics of the intestinal microbiome of HD patients with or without enterocolitis. During routine or emergency surgery, we collected 35 intestinal content samples from five patients with HAEC and eight HD patients, including three HD patients with a history of enterocolitis who were in a HAEC remission (HAEC-R) phase. Using Illumina-MiSeq high-throughput sequencing, we sequenced the V4 region of bacterial 16S rRNA, and operational taxonomic units (OTUs) were defined by 97% sequence similarity. Principal coordinate analysis (PCoA) of weighted UniFrac distances was performed to evaluate the diversity of each intestinal microbiome sample. The microbiota differed significantly between the HD patients (characterized by the prevalence of *Bacteroidetes*) and HAEC patients (characterized by the prevalence of *Proteobacteria*), while the microbiota of the HAEC-R patients was more similar to that of the HAEC patients. We also observed that the specimens from different intestinal sites of each HD patient differed significantly, while the specimens from different intestinal sites of each HAEC and HAEC-R patient were more similar. In conclusion, the microbiome pattern of the HAEC-R patients was more similar to that of the HAEC patients than to that of the HD patients. The HD patients had a relatively distinct, more stable community than the HAEC and HAEC-R patients, suggesting that enterocolitis may either be caused by or result in a disruption of the patient’s uniquely adapted intestinal flora. The intestinal microbiota associated with enterocolitis may persist following symptom resolution and can be implicated in the symptom recurrence.

## Introduction

Hirschsprung’s disease (HD), a developmental disorder of the enteric nervous system, is characterized by the congenital absence of parasympathetic intrinsic ganglion cells in the submucosal and myenteric plexuses of a variable portion of the distal gut, with proximal extension and functional intestinal obstruction [1,2]. The incidence of HD in live births is estimated to be roughly 1 in 5,000 [2]. HD patients experience delayed passage of meconium, neonatal intestinal obstruction, chronic constipation, and abdominal distension. Although HD can be treated surgically in most cases using a minimally invasive approach, and is often resolved without colostomy [1], the incidence rate of a severe life-threatening complication Hirschsprung-associated enterocolitis (HAEC) remains as high as 17 to 50% [3,4]. HAEC is an inflammatory colitis that causes distension, diarrhea, and fever and can lead to bacterial translocation, sepsis, and death. HAEC remission is characterized by resolution of clinical symptoms of inflammation and by a return of circulating levels of inflammatory factors to the normal range [5]. Over the past 20 years, due to early diagnosis, rectal decompression, appropriate vigorous resuscitation, and antibiotic therapy, the mortality rate from HAEC has decreased from 30% to 1–10%. However, HAEC remains the leading cause of morbidity and the most common cause of death in infants and children with HD [3,4].

Although the etiology and mechanisms of the HAEC pathogenesis have not been determined, abnormalities in the intestinal microbiome have been implicated in the development of HAEC. Bacteria and viruses, including Clostridium difficile and rotavirus, have been associated with enterocolitis [6–9]. However, the relationship between Clostridium species and the development of HAEC remains controversial [9,10].

Furthermore, HAEC patients are prone to recurrent episodes, and the recurrence rate ranges from 5.2% to 56% [4]. Recurrence may be attributed to sustained histopathological alterations in the intestinal mucosae or to immunodeficiencies or dysfunctions of the intestinal immune system [11]. Recently, De Filippo et al. [5] assessed 15 stool specimens from a three-year-old patient with enterocolitis using amplified ribosomal DNA restriction analysis (ARDRA) and suggested that the occurrence and recurrence of enterocolitis may be associated with a specific distribution of intestinal flora, which is influenced by the use of antibiotics.

Attempts to characterize the role of intestinal microbiota in the physiological mechanisms underlying HAEC have been hampered by the complexity of the intestinal flora and by the limitations of cultivation-based techniques, which can culture less than 15% of intestinal bacterial species. While traditional microbiological techniques have been focused on studying individual potential pathogens, the development of high-throughput sequencing and metagenomics approaches has made it possible to analyze the intestinal microbiota comparatively and precisely and allowed the distribution patterns of intestinal microbiomes to be characterized and compared [12–15]. In a small preliminary study, we have previously applied the Illumina-MiSeq high-throughput sequencing platform to study the structure of intestinal microbiota in HD infants with and without enterocolitis [16]. Here, we sought to expand this approach to a larger population, to explore the characteristics of the intestinal microbiome patterns in HAEC remission (HAEC-R) patients, and to investigate the hypothesis that the intestinal microbiome differs significantly among patients with HD, depending on whether they have enterocolitis or not.

## Methods

### Subjects and specimens

This study cohort comprised 13 patients who were treated at Shanghai Children’s Medical Center between April 20, 2012 and August 8, 2013. The ethical approval (SCMCIRB-K2012022) was obtained from the Joint Ethics Committee of the Shanghai Children’s Medical Center, which is affiliated with Shanghai Jiaotong University. Detailed written informed consent was obtained from the parents of each patient. All patients were diagnosed with HD based on pathology. Five had no history of enterocolitis, while eight experienced at least one episode of HAEC, based on the HAEC scoring system described by Pastor et al. [17]. The patients had not received probiotics or antibiotics for at least five days prior to the collection of the intestinal contents. The patients’ demographic and clinical characteristics are listed in Table 1.

**Table 1:** Patient Demographic and Clinical Characteristics of Hirschsprung’s Disease with or without Enterocolitis.

Patients No.	Sample No.	Gender	Age(months)	Diagnosis	Site	Grouping
1	1	male	2	HAEC	transverse colon	proximal
	2				appendix	proximal
	3				ileum	proximal
	4				rectum	distal
2	5	male	6	HAEC	ascending colon	proximal
	6				sigmoid colon	distal
	7				appendix	proximal
	8				rectum	distal
3	9	female	7	HD	descending colon	distal
	10				transverse colon	proximal
4	11	male	12	HD	transverse colon	proximal
	12				appendix	proximal
	13				rectum	distal
5	14	male	3	HAEC	sigmoid colon	distal
	15				descending colon	distal
	16				ascending colon	proximal
	17				appendix	proximal
6	18	female	0.3	HAEC	sigmoid colon	distal
7	19	male	12	HD*	anus	distal
	20				sigmoid colon	proximal
8	21	female	48	HAEC	transverse colon	distal
	22				ileum	proximal
	23				cecum	distal
	24				sigmoid colon	distal
9	25	female	12	HD	descending colon	proximal
	26				sigmoid colon	distal
10	27	female	24	HD*	sigmoid colon	proximal
	28				anus	distal
11	29	female	10	HD	anus	distal
	30				ileum	proximal
12	31	male	21	HD	rectum	distal
	32				sigmoid colon	proximal
	33				transverse colon	proximal
13	34	male	5	HD*	anus	distal
	35				sigmoid colon	proximal

The patients were divided into two groups, HAEC (n = 5) and HD (n = 8), according to the presence of enterocolitis at the time of sampling. Patients No. 7, 10, and 13, marked as HD*, had a history of enterocolitis but did not have any symptoms of enterocolitis when sampled. They then formed a subgroup of HD termed the HAEC-R remission (HAEC-R) group. According to the pathological diagnosis, “proximal” means samples collected from the intestine with ganglion cells, and “distal” means samples collected from the intestine without ganglion cells.

### Specimen collection

Patients’ intestinal contents were sampled during surgery from one to four sites within the intestine of each patient. The specimens were taken from the ileum, cecum, appendix, ascending colon, transverse colon, descending colon, sigmoid colon, rectum, and anus as indicated (Table 1). The intestinal content specimens were kept on dry ice immediately after the collection and placed within 30 min to a −80°C freezer, where they were stored without any additive until analysis.

### DNA extraction

DNA was isolated from each specimen using the PowerSoil DNA isolation kit (MO BIO Laboratories, Carlsbad, CA, USA) according to the Human Microbiome Initiative guidelines. The DNA samples were stored at −80°C.

### 16S rDNA sequencing

The V4 region of the bacterial 16S rRNA gene was amplified from each total DNA sample using bacterial/archaeal primers 515F (5′-GTGCCAGCMGCCGCGGTAA-3′) and 806R (5′-GGACTACHVGGGTWTCTAAT-3′), as previously described by Caporaso et al. [18]. Polymerase chain reaction (PCR) was performed in a volume of 25 μL using the TaKaRa ExTaq enzyme mixture (Takara Bio, Inc., Shiga, Japan) and 200 ng of fecal DNA. The cycling conditions were as follows: initial denaturation at 94°C for 3 min, followed by 25 cycles of 94°C for 45 s, 50°C for 1 min, and 72°C for 1min, and a final extension at 72°C for 10 min. The PCR products were stored at 4°C. Equal quantities of each sample were pooled, and the PCR products were gel-purified and sequenced by MiSeq (Illumina) according to the manufacturer’s protocol. Reads were demultiplexed using the Illumina software and separate FASTQ files were generated for each specimen and deposited to Sequence Read Archive NCBI under the BioProject accession PRJNA322917.

### Data analysis

Sequences were processed using the QIIME pipeline (1.8.0) [19]. Briefly, quality filtering uses the following rules: (1) no mismatches are allowed in the barcode; (2) no N bases are allowed in the reads; (3) a read with three or more consecutive low-quality base calls is trimmed; (4) trimmed reads should be > 0.75 of the original read length. The quality-filtered reads were clustered into operational taxonomic units (OTUs) at a 97% identity level. Taxonomies were assigned with the UCLUST algorithm against the Greengenes (Version 13.8) reference sequences. Alpha diversity was calculated using the observed species metrics. To calculate beta diversity (weighted UniFrac distance), 8,000 sequences were randomly selected from each sample [20]. Statistical significance of the factors potentially contributing to compositional differences between samples was examined using the non-parametric permutation analysis of similarity (ANOSIM), which was performed using QIIME (version 1.8.0). LEfSe was used for detecting the differences in the abundance of bacterial species between groups. We used the default settings in LEfSe (alpha value of 0.05 for the Kruskal–Wallis and pairwise Wilcoxon tests and 2.0 for the threshold on the logarithmic linear discriminant analysis score).

## Results

### A deeper look at the microbiome in HD and HAEC patients

In this study, we characterized the intestinal microbiota of 13 patients between 10 days and 48 months of age, with or without Hirschsprung’s disease associated enterocolitis, using a 16S rRNA gene-tag sequence-based method. Samples of intestinal contents were collected during surgery from one to four sites within the intestine of each patient. The patients were divided into the HAEC (n = 5) and HD (n = 8) groups according to the presence of enterocolitis at the time of sampling. In the HD group, three patients had a medical history of enterocolitis but were in remission when sampled; they were thus assigned to a subgroup termed the HAEC-R group (Table 1).

We sequenced 16S rDNA from a total of 35 intestinal content specimens, 17 specimens from the HAEC patients and 18 specimens from the patients with HD, including 6 specimens from the HAEC-R patients. A mean of 67,165 reads (ranging between 8,006 and 175,055 reads) were generated using the Illumina-MiSeq platform for each sample.

### Sequence-based relationships between specimens

**Fig 1:**
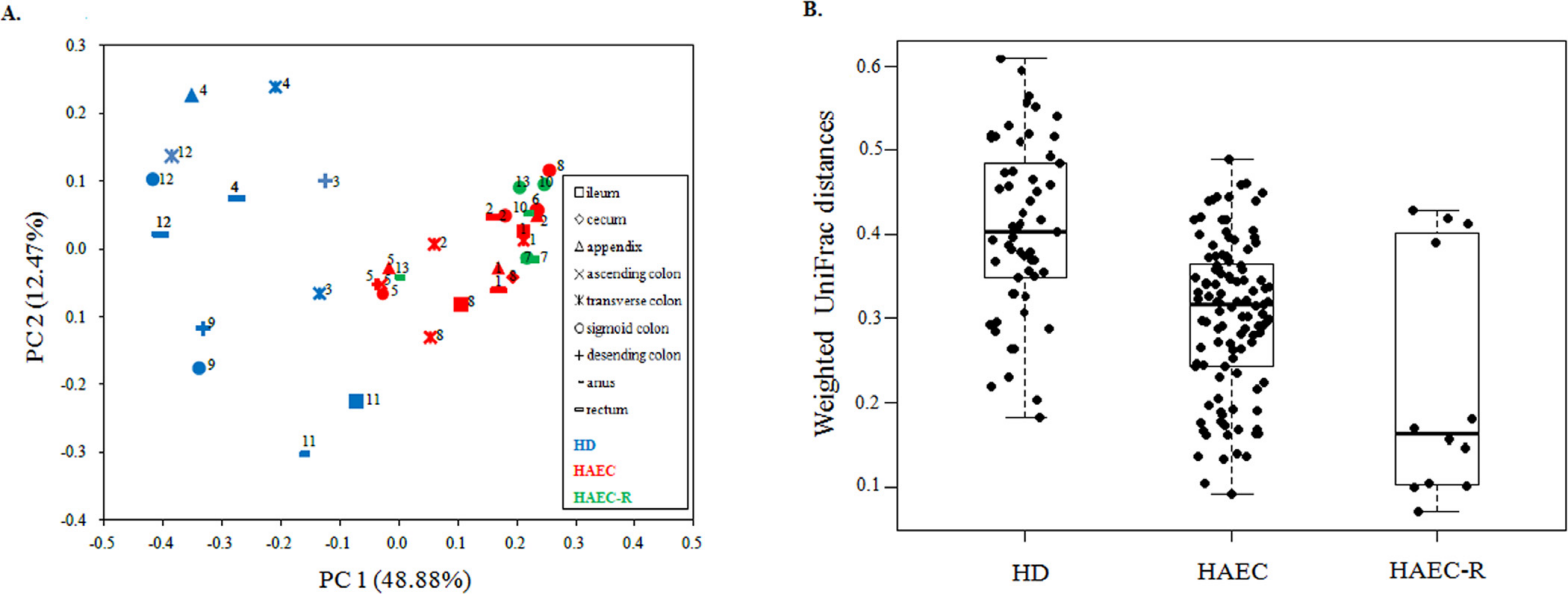
The intestinal microbiome pattern of the HAEC-R patients is more similar to that of the HAEC patients than to that of the HD patients, and the HD patients have a relatively distinct, stable community compared with those of the HAEC and HAEC-R patients. (A) Principal coordinate analysis (PCoA) of unweighted UniFrac distances for patients with different diagnoses. PC1, first principal coordinate; PC2, second principal coordinate. Numbers represent the patient ID numbers. The phylogenetic composition of the fecal microbiota in the HAEC-R patients was more similar to that of the HAEC patients (ANOSIM, *p* > 0.05) than to that of the HD patients (ANOSIM, *p* < 0.01). (B) The weighted UniFrac distances between the most distant samples of each patient. The microbiota of each HAEC or HAEC-R patient is more similar between the sample sites (Student’s *t*-test, *p* > 0.05) than that of each HD patient (Student’s *t*-test, *p* < 0.01).

We performed a principal coordinate analysis (PCoA) of weighted UniFrac distances to compare the diversity of each intestinal microbiome sample. The first and second principal coordinates accounted for 48.88% and 12.47% of the intersample variance, respectively. The phylogenetic compositions of the intestinal microbiotas were found to differ significantly between the HD and HAEC patients (Fig 1A; ANOSIM, *p* < 0.01; S1 Table), consistent with previous studies [17,19]. The phylogenetic composition of the fecal microbiota from the HAEC-R patients (patients 7, 10, and 13) was more similar to that of the HAEC patients (Fig 1A; ANOSIM, *p* = 0.31; S1 Table) than to that of the HD patients (Fig 1A; ANOSIM, *p* < 0.01; S1 Table), indicating that the pattern of intestinal microbes associated with enterocolitis may persist following the resolution of symptoms and may be implicated in the recurrence of symptoms.

The distances between the most distant samples of each patient using coordinates from the UniFrac matrix were also calculated. We observed that the specimens from different intestinal sites in each HD patient differed significantly (Fig 1B; Student’s t-test, *p* < 0.01; S2 Table), while the specimens from different intestinal sites in each HAEC and HAEC-R patients were more similar (Fig 1B; Student’s t-test, *p* = 0.08; S2 Table). These observations suggest that HD patients have a relatively distinct, stable community compared with HAEC and HAEC-R patients. These results may suggest that enterocolitis is either caused by or results in a disruption of the patient’s uniquely adapted intestinal flora.

### Taxonomic variation

**Fig 2:**
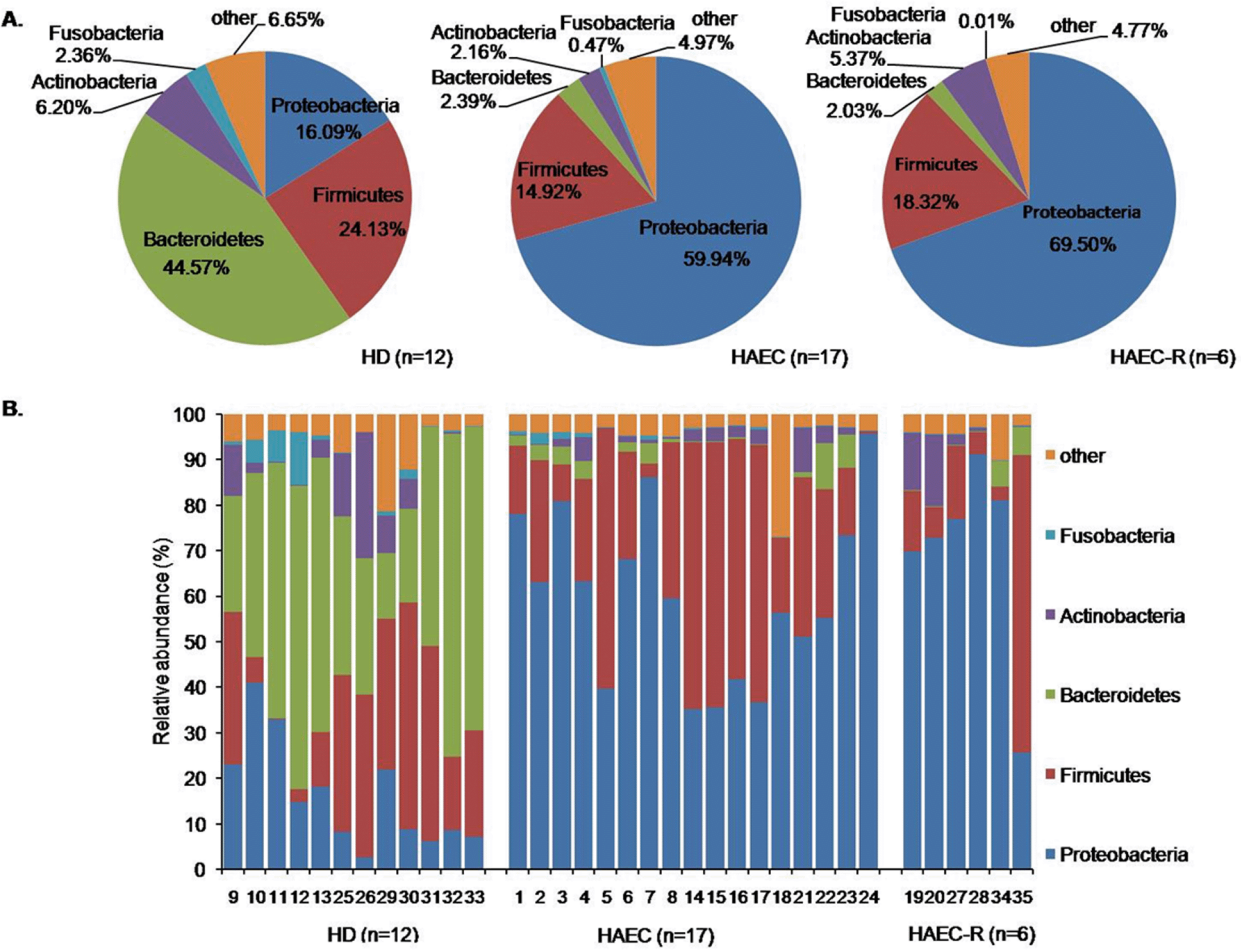
Relative abundance of phyla in intestinal microbiomes. Relative abundance of phyla in the intestinal microbiomes of the HD, HAEC, and HAEC-R patients. (A) The pie charts show the average relative abundance of five major phyla. (B) The histograms demonstrate the bacterial composition of the stool samples at the phylum level. The X-axis shows the sample numbers. The colors assigned to each of the five detected phyla are shown at the right side.

In order to identify specific taxa responsible for the variation in intestinal microbiotas, the relative abundance of OTUs was determined at the phylum, class, order, family, and genus levels. The 16S rRNA gene OTUs were defined by the 97% sequence similarity. The intestinal microbiota of the HD patients was characterized by high levels of *Bacteroidetes* (45%), *Firmicutes* (24%), and *Proteobacteria* (16%). In contrast, the most abundant phylum detected in the HAEC patients was *Proteobacteria* (60%), followed by *Firmicutes* (30%). The microbiota of the HAEC-R patients was similar to that of the HAEC patients in that it was characterized by the abundance of *Proteobacteria* (70%) and *Firmicutes* (18%) (Fig 2A and 2B; S1 Appendix) and showed an increased abundance of Proteobacteria and a reduced abundance of *Bacteroidetes*.

**Fig 3:**
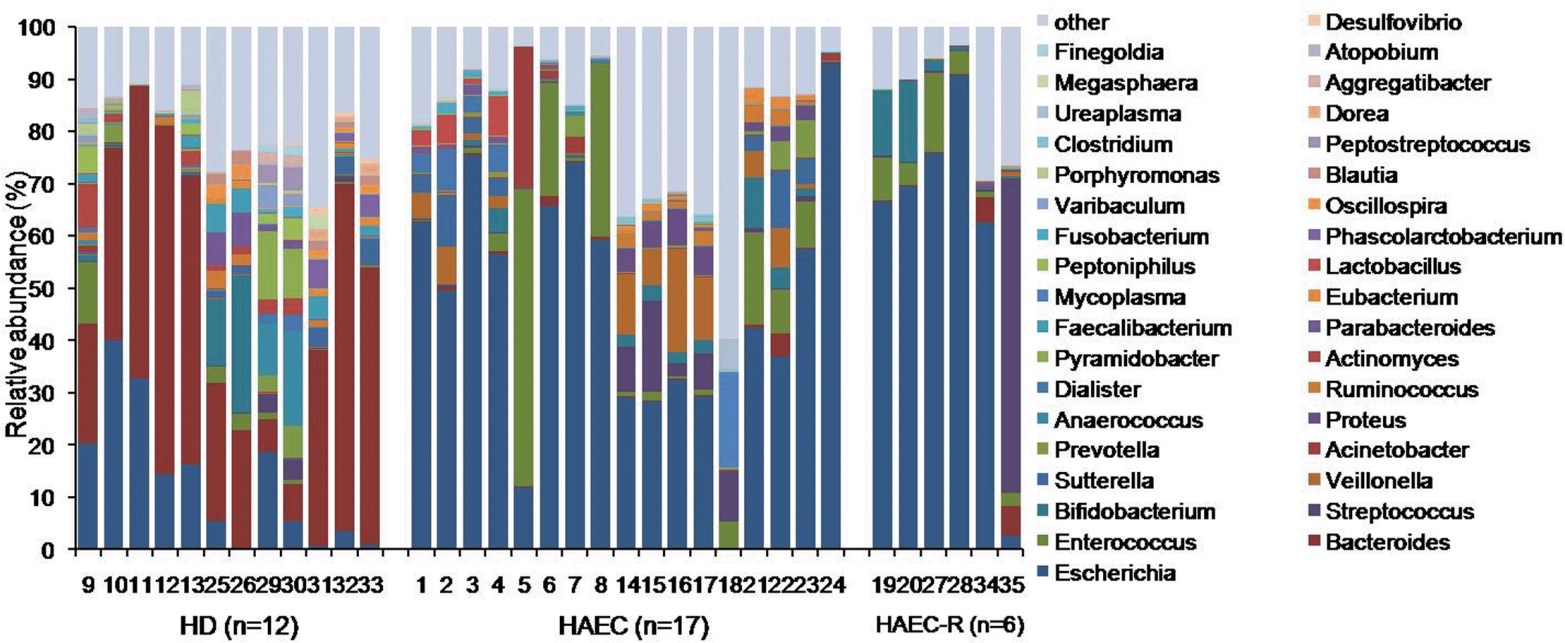
Relative abundance of genera in intestinal microbiomes. While *Escherichia* was the most prominent genus detected in the HAEC (47%) and HAEC-R (61%) patients, *Bacteroides* (38%) was the most prominent genus in the HD patients.

*Escherichia* was the most prominent genus detected in the HAEC and HAEC-R patients, representing 47% and 61% of all OTUs, respectively, followed by *Bacteroides* (10%), *Veillonella* (5%), and *Acinetobacter* (2%) in the HAEC patients and by *Streptococcus* (10%) and *Enterococcus* (6%) in the HAEC-R patients. In contrast, *Bacteroides* (38%) was the most prominent genus in the HD patients, followed by *Escherichia* (13%) and *Bifidobacterium* (3.5%) (Fig 3; S1 Appendix). At the genus level, both HAEC and HAEC-R groups showed a decreased abundance of *Bacteroides* and an increased abundance of *Escherichia* compared to the HD group.

**Fig 4:**
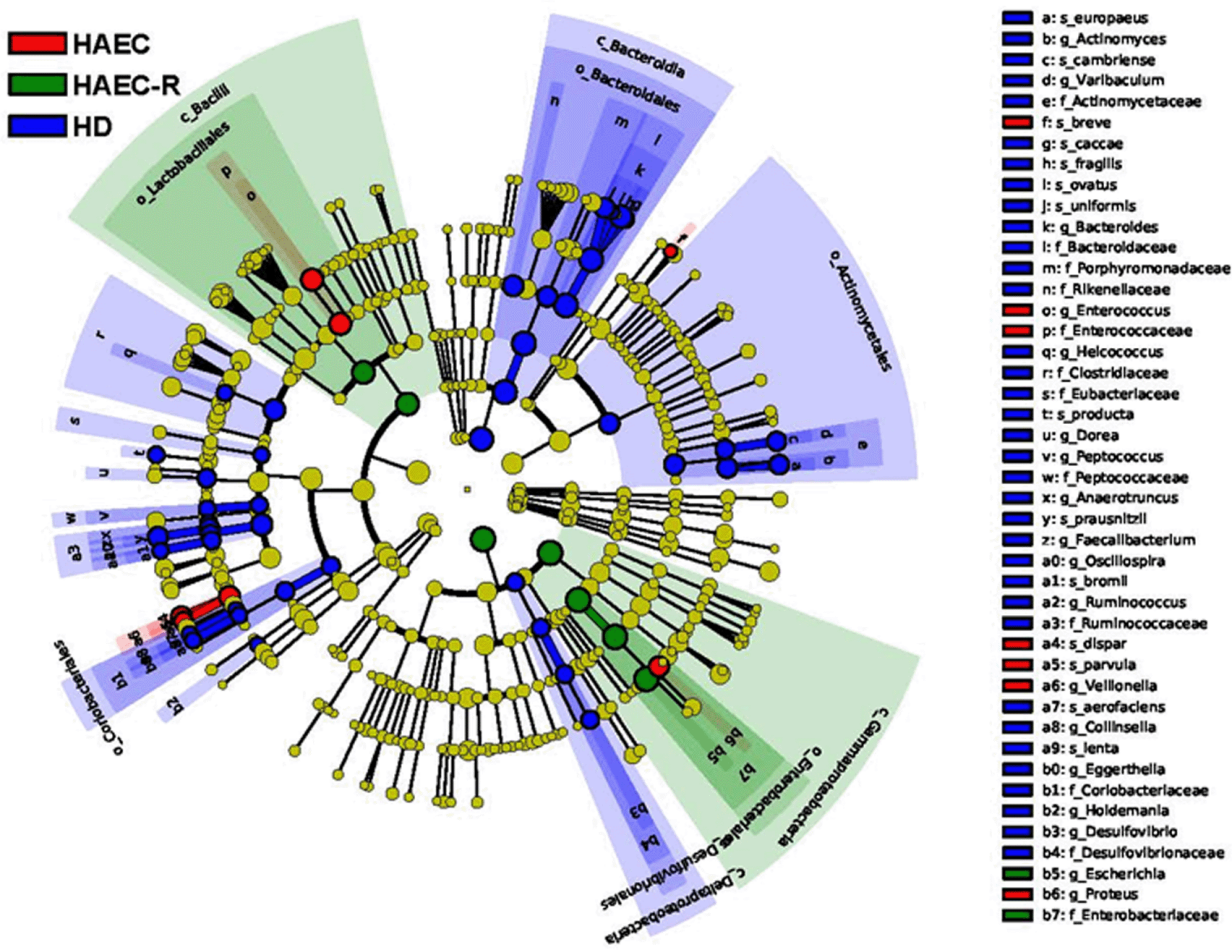
The cladogram generated by the LEfSe software illustrates the taxa with different abundance among the three groups (HD, HAEC, and HAEC-R). Different taxa are colored according to the most abundant class, and the size of the circle corresponds to the relative abundance of the taxon.

**Fig 5:**
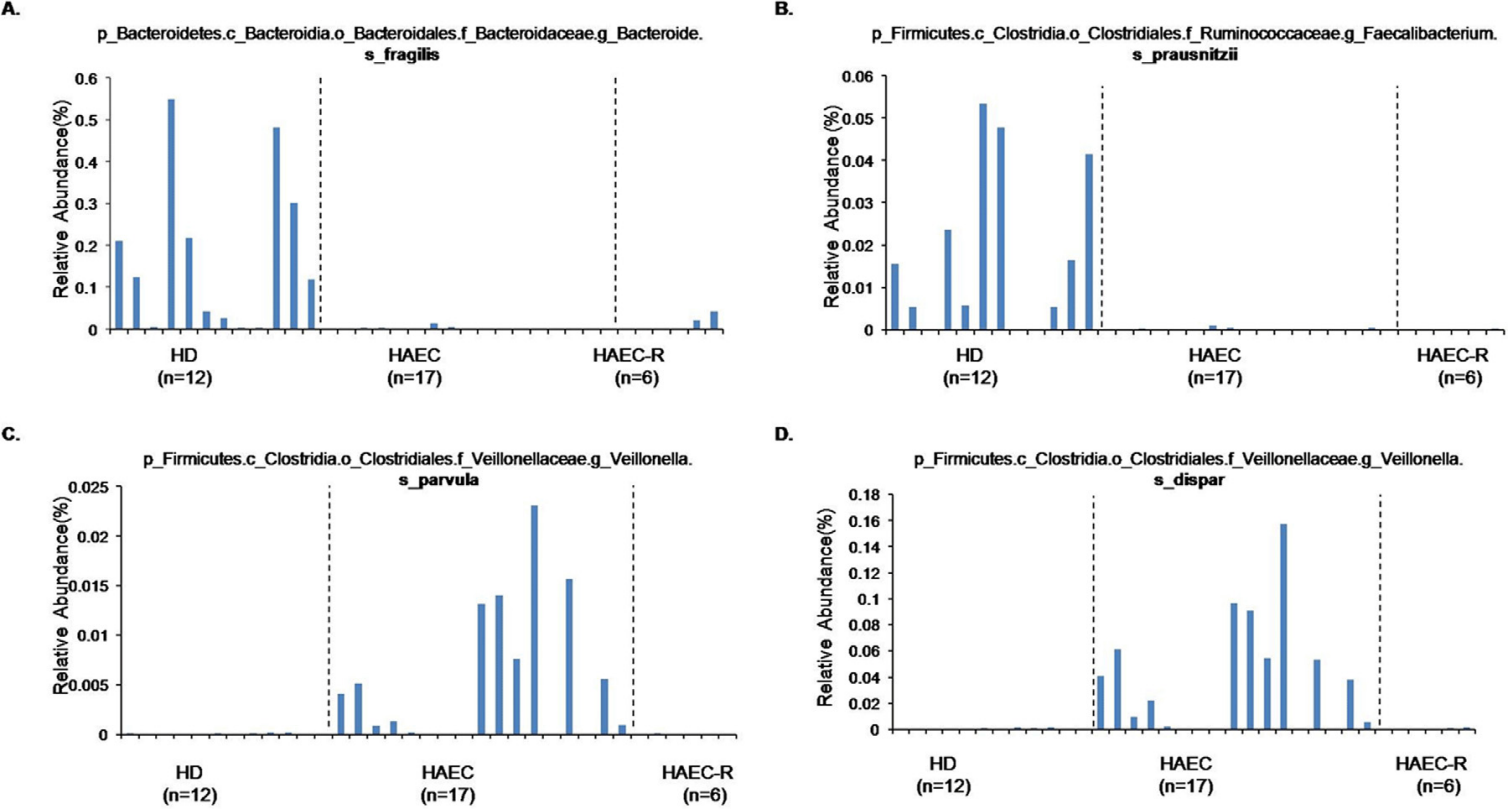
Relative abundance of four species those are most different in the abundance in the guts of the HD, HAEC, and HAEC-R patients. Relative abundance of *Bacteroides fragilis* (A), *Faecalibacterium prausnitzii* (B), *Veillonella parvula* (C), and *Veillonella dispar* (D) in the intestines of five HD patients, five HAEC patients, and three HAEC-R patients.

The cladogram (Fig 4; S1 Dataset) illustrates the taxa that differed in abundance among the three patient groups. We also observed that OTU1308 and OTU3822, which were tentatively annotated as *Bacteroides fragilis* and *Faecalibacterium prausnitzii*, were detected almost exclusively in the HD patients, while OTU1559 and OTU4284, annotated as *Veillonella parvula* and *V*. *dispar*, were almost exclusively found in the samples from the HAEC patients (Fig 5; S1 Dataset).

## Discussion

HAEC is characterized by an acute inflammation of the crypts and intestinal epithelium mucosa [21,22], and the intestinal microbiota has been implicated in a variety of inflammatory gut disorders [23], including HAEC [4–9,16,17]. While genetic determinants of HD have also been sought [24], several studies have suggested that bacterial overgrowth or the presence of specific bacterial or viral pathogens, such as *C*. *difficile* or rotavirus, is associated with enterocolitis [6–9]. However, no specific pathogen has been implicated in the etiology of HAEC. Hence, the shift from the search for the single pathogen to the study of HD-related bacterial community is logical and can provide a new insight into etiology of HAEC.

In this study, we compared intestinal microbiotas of HD, HAEC, and HAEC-R patients using Illumina-MiSeq high-throughput sequencing and found that the microbiotas of the HD and HAEC patients differed significantly, while that of the HAEC-R patients was more similar to that of the HAEC patients (Fig 1A; S1 Table). The intestinal microbiota of the HD patients was characterized by high levels of *Bacteroidetes*, *Firmicutes*, and *Proteobacteria*. In contrast, the most abundant phylum detected in the HAEC and HAEC-R patients was *Proteobacteria*, followed by *Firmicutes* (Fig 2A; S1 Appendix). Additionally, we observed that the specimens from different intestinal sites differed significantly in each HD patient, while the specimens from different intestinal sites were more similar in each HAEC and HAEC-R patient (Fig 1B; S2 Table).

The reported incidence of HAEC ranges from 17 to 50% [3,4]. Preoperative HAEC has been reported to occur in 5.7–50% of patients, and even after surgery, the incidence of enterocolitis has been reported to range from 2 to 35%. Several factors, such as trisomy 21, a family history, long-segment Hirschsprung’s disease, and previous episodes of HAEC, can increase the risk of HAEC. Additionally, each episode of HAEC may increase the risk of future HAEC, regardless of the therapy, and the incidence of recurrent HAEC ranges from 5.2 to 56% [4]. Reding et al. [25] reported 12 cases of preoperative HAEC, four of which had recurrent enterocolitis in the postoperative period. A retrospective study reported that among 168 HD patients, 57 patients developed 119 episodes of HAEC within 18 years [26]. In this study, we characterized the intestinal microbiota of 13 HD patients. Five of them experienced enterocolitis at the time of sampling, and three had a medical history of enterocolitis but were in remission when sampled. The microbiota of the HD patients differed significantly from that of the patients with enterocolitis, while the microbiota of the patients in the enterocolitis remission phase was more similar to that of the patients with enterocolitis than to the microbiota of those without a history of enterocolitis.

Using ARDRA, De Filippo et al. [5] have recently characterized 15 fresh stool samples from an HD patient followed up longitudinally during three episodes and remission phases of enterocolitis. The authors found that the microbiota differed significantly between the periods of enterocolitis and remission during four episodes of enterocolitis. Nonetheless, in this study, we found that in periods of remission the gut microbiota still bore hallmarks of enterocolitis since it shared more similarity with the microbiota of the patients currently experiencing enterocolitis than with that of the patients who had never experienced enterocolitis. Our results are further supported by the findings of Frykmann et al. [27] who compared the fecal bacterial and fungal communities of HD children, nine of which experienced enterocolitis and nine that did not, and found enterocolitis to be associated with a distinct microbiota, in particular an altered Candida community. Additionally, it was found, based on a previous long-term follow-up of 259 patients, that even after one episode of HAEC, intestinal dysfunction worsened more severely in HD patients with a history of enterocolitis than in those without a prior history of enterocolitis [28], further indicating that long-term changes in the gut are associated with enterocolitis.

Strikingly, in this study we found that while the gut microbiotas differed significantly between the HD (characterized by high levels of *Bacteroidetes*) and HAEC patients (characterized by high levels of *Proteobacteri*a), the microbiota of the HAEC-R patients was more similar to that of the HAEC patients (Fig 1A; S1 Table). These results support the previous studies suggesting that the gut microbiota differs significantly in patients who experience enterocolitis [5,17]. Our findings imply that the intestinal flora of HAEC patients is altered in the long term, and HAEC-R patients with a history of enterocolitis, even in remission, have a microbiota more similar to that of patients currently experiencing enterocolitis than to that of HD patients who have not experienced enterocolitis. These findings support the previous reports indicating that the risk of the HAEC recurrence is extremely high [4,11,21]. Therefore, we hypothesize that even when the symptoms of enterocolitis are resolved, intestinal microbiome remains characteristic of enterocolitis and potentially contributes to the enterocolitis recurrence. Recurrent emergence of HAEC may be caused by pathological changes to the tissue or by immune responses of the gut mucosa [11]. Histologically, enterocolitis is characterized by infiltration of neutrophils into the crypts and retention of mucus leading to crypt dilatation and intestinal wall inflammation, which can contribute to the loss of epithelial barrier function. Consequently inflammation can worsen and persist, initiating a vicious cycle of perpetual inflammation [29]. This may explain recurrent enterocolitis. Even after surgery, the protective mucus barrier of the ganglionated bowel is impaired, which may contribute to the occurrence of postoperative HAEC and recurrent episodes of enterocolitis [21]. The capacity of the intestinal immune system to defend the gut from microbial pathogens requires that immunologic homeostasis is maintained. However, HAEC patients have a significant deficiency in secretory immunoglobulin A and are hypersensitive to microbial antigens, which may contribute to the recurrence of HAEC [30]. In the future, we will further study the relationship between the intestinal dysbiosis and immunological injury.

In this study we also found that microbiota from different intestinal sites in each HD patient differed significantly, whilst microbiota from different intestinal sites in each HAEC and HAEC remission patient are more similar (Fig 1B; S2 Table). That means that HD patients contained a relatively distinct community of bacteria in different sites of the intestine, however, in HAEC and HAEC-R patients different sites of the intestine possessed a similar microbiota. It may indicate that the microbiota in the different sites of intestine of enterocolitis patients is losing the site-specific properties, and falls into the similar illness-related disordered pattern. Hence, the administration of probiotics, recently proposed to reduce incidence and severity of HAEC [31], looks as an appropriate way of clinical intervention. Hence, failure to demonstrate a reduced incidence of HAEC in the single prospective, randomized, double-blinded, placebo-controlled, multicenter trial [32], could be attributed to the cohort size, the selected probiotic formulation, the dosage or the route of administration, rather than a faulty concept.

## Conclusions

In this study, we collected 35 samples of intestinal contents from five HAEC patients, five HD patients, and three HAEC-R patients and found that the constitution of intestinal microbiomes differed significantly between the HD and HAEC patients. The HAEC-R patients had an intestinal microbiome more similar to that of the HAEC patients than to that of the HD patients. Furthermore, the intestinal microbiota of the HD patients without enterocolitis was relatively distinct and more stable than that of the HAEC and HAEC-R patients. This observation may indicate that enterocolitis either causes or results from a loss of the patient’s uniquely adapted intestinal flora and thus suggests the presence of a bacterial community that can predispose patients to the HAEC recurrence. We conclude that identifying bacterial communities responsible for this pathological phenotype may allow the development of preventative treatments. However, since the numbers of patients and samples in this study were limited and the study was cross-sectional in design, our conclusions are limited. Further, larger longitudinal studies will allow to study the alterations in the intestinal microbiota during the emergence and resolution of HAEC.

## Supporting Information

S1 Appendix Relative abundance of dominant bacterial in feces of HD, HAEC and HAEC-R at the phylum, class, order, family and genus level. (ZIP)

S1 Dataset The taxa that differed in abundance among the three groups. (XLSX)

S1 Table The values of the first principal coordinate (PC1) and PC2 of each fecal sample. (DOCX)

S2 Table The weighted unifrac distances of the specimens from different intestinal sites in each group. (DOCX)
