# Supplementary material for: Characterization of Intestinal Microbiomes of Hirschsprung’s Disease Patients with or without Enterocolitis Using Illumina-MiSeq High-Throughput Sequencing
Source: PLoS One. 2016 Sep 7;11(9):e0162079. doi: 10.1371/journal.pone.0162079 (PMC5014423; doi:10.1371/journal.pone.0162079)
Supplement: S1 Table — (DOCX) [file pone.0162079.s003.docx]

**S1 Table. The values of the first principal coordinate (PC1) and PC2 of each fecal sample.**

| **People No.** | **Sample_No** | **Diagnosis** | **PC1** | **PC2** |
| --- | --- | --- | --- | --- |
| 1 | 1 | HAEC | 0.211179167 | 0.011898293 |
|  | 2 | HAEC | 0.168361113 | -0.028030346 |
|  | 3 | HAEC | 0.211503419 | 0.026050094 |
|  | 4 | HAEC | 0.168776148 | -0.061325478 |
| 2 | 5 | HAEC | 0.059920427 | 0.006773013 |
|  | 6 | HAEC | 0.179741143 | 0.048823349 |
|  | 7 | HAEC | 0.234110768 | 0.049542342 |
|  | 8 | HAEC | 0.163084087 | 0.04615552 |
| 3 | 9 | HD | -0.124976974 | 0.099983821 |
|  | 10 | HD | -0.134474675 | -0.065909274 |
| 4 | 11 | HD | -0.209368842 | 0.238765343 |
|  | 12 | HD | -0.351315721 | 0.22676641 |
|  | 13 | HD | -0.274744034 | 0.074595067 |
| 5 | 14 | HAEC | -0.027587797 | -0.066595135 |
|  | 15 | HAEC | -0.037175786 | -0.052655154 |
|  | 16 | HAEC | -0.029573875 | -0.052315174 |
|  | 17 | HAEC | -0.017022966 | -0.027641199 |
| 6 | 18 | HAEC | 0.23396259 | 0.056871181 |
| 7 | 19 | HAEC-R | 0.207518226 | -0.015686054 |
|  | 20 | HAEC-R | 0.216756904 | -0.013264528 |
| 8 | 21 | HAEC | 0.053184413 | -0.130604162 |
|  | 22 | HAEC | 0.104031436 | -0.081767132 |
|  | 23 | HAEC | 0.193067535 | -0.042208293 |
|  | 24 | HAEC | 0.255110936 | 0.1156792 |
| 9 | 25 | HD | -0.331851554 | -0.11697003 |
|  | 26 | HD | -0.338895583 | -0.175883555 |
| 10 | 27 | HAEC-R | 0.246237413 | 0.094921556 |
|  | 28 | HAEC-R | 0.214039085 | 0.05225702 |
| 11 | 29 | HD | -0.1651587 | -0.30281895 |
|  | 30 | HD | -0.072045615 | -0.225423437 |
| 12 | 31 | HD | -0.404220562 | 0.021378775 |
|  | 32 | HD | -0.417426691 | 0.10268455 |
|  | 33 | HD | -0.385392414 | 0.13702363 |
| 13 | 34 | HAEC-R | -0.003822357 | -0.041767715 |
|  | 35 | HAEC-R | 0.204469334 | 0.090696451 |
