# Supplementary material for: Characterization of Intestinal Microbiomes of Hirschsprung’s Disease Patients with or without Enterocolitis Using Illumina-MiSeq High-Throughput Sequencing
Source: PLoS One. 2016 Sep 7;11(9):e0162079. doi: 10.1371/journal.pone.0162079 (PMC5014423; doi:10.1371/journal.pone.0162079)
Supplement: S2 Table — (DOCX) [file pone.0162079.s004.docx]

**S2 Table. The weighted unifrac distances of the specimens from different intestinal sites in each group.**

| **HD** | **HAEC** | **HAEC-R** |
| --- | --- | --- |
| 0.412683021 | 0.33804739 | 0.101039889 |
| 0.394619816 | 0.418780231 | 0.099972778 |
| 0.418476317 | 0.288487081 | 0.155856835 |
| 0.370262843 | 0.279839787 | 0.146948342 |
| 0.355157617 | 0.340612388 | 0.104355483 |
| 0.378861069 | 0.234786809 | 0.391293706 |
| 0.383110545 | 0.28886451 | 0.070796975 |
| 0.409282159 | 0.23017453 | 0.419877893 |
| 0.229353565 | 0.3023661 | 0.179844191 |
| 0.202902451 | 0.37463243 | 0.413493826 |
| 0.306849385 | 0.2967264 | 0.16925114 |
| 0.264035019 | 0.319982075 | 0.429352807 |
| 0.287208671 | 0.445862094 |  |
| 0.397185727 | 0.403660595 |  |
| 0.404204729 | 0.491066537 |  |
| 0.493940055 | 0.449659197 |  |
| 0.325944004 | 0.271269413 |  |
| 0.374802125 | 0.204779762 |  |
| 0.379479075 | 0.308445546 |  |
| 0.474094732 | 0.189837461 |  |
| 0.387140847 | 0.354012159 |  |
| 0.348415871 | 0.305485569 |  |
| 0.330023734 | 0.340588988 |  |
| 0.292185588 | 0.296985488 |  |
| 0.181742291 | 0.330126496 |  |
| 0.218947005 | 0.139458423 |  |
| 0.329405793 | 0.160514363 |  |
| 0.284415616 | 0.167354738 |  |
| 0.263542061 | 0.320405122 |  |
| 0.516377219 | 0.136951617 |  |
| 0.486247613 | 0.092497612 |  |
| 0.528346383 | 0.166414409 |  |
| 0.518284147 | 0.242493949 |  |
| 0.539805158 | 0.261711613 |  |
| 0.516684897 | 0.243852284 |  |
| 0.296150519 | 0.175659065 |  |
| 0.368081072 | 0.281083349 |  |
| 0.369815705 | 0.245639386 |  |
| 0.455161403 | 0.302306011 |  |
| 0.356379722 | 0.216099253 |  |
| 0.426593831 | 0.191530246 |  |
| 0.519769634 | 0.272522758 |  |
| 0.609713747 | 0.166678169 |  |
| 0.555858274 | 0.188776868 |  |
| 0.594600094 | 0.196477728 |  |
| 0.407627306 | 0.326830341 |  |
| 0.476534691 | 0.137342816 |  |
| 0.466328856 | 0.263612701 |  |
| 0.564763879 | 0.299113782 |  |
| 0.451168185 | 0.265619394 |  |
| 0.551512805 | 0.323480139 |  |
| 0.35092044 | 0.290688664 |  |
| 0.441251637 | 0.291540616 |  |
| 0.459981637 | 0.269554911 |  |
| 0.509178826 | 0.314317184 |  |
| 0.458024144 | 0.282760052 |  |
| 0.514303562 | 0.40066656 |  |
|  | 0.374636052 |  |
|  | 0.418626426 |  |
|  | 0.397122822 |  |
|  | 0.442853305 |  |
|  | 0.41854335 |  |
|  | 0.461572244 |  |
|  | 0.439700894 |  |
|  | 0.346143271 |  |
|  | 0.373905458 |  |
|  | 0.34097978 |  |
|  | 0.319586497 |  |
|  | 0.163088873 |  |
|  | 0.25255939 |  |
|  | 0.133421627 |  |
|  | 0.163370982 |  |
|  | 0.177825973 |  |
|  | 0.29432928 |  |
|  | 0.104115489 |  |
|  | 0.223188289 |  |
|  | 0.184346137 |  |
|  | 0.243501401 |  |
|  | 0.172777142 |  |
|  | 0.160543569 |  |
|  | 0.359359671 |  |
|  | 0.333223412 |  |
|  | 0.393770404 |  |
|  | 0.360306831 |  |
|  | 0.376757352 |  |
|  | 0.351223537 |  |
|  | 0.396655351 |  |
|  | 0.372960184 |  |
|  | 0.4449246 |  |
|  | 0.421662752 |  |
|  | 0.459756732 |  |
|  | 0.440799293 |  |
|  | 0.36263824 |  |
|  | 0.336192526 |  |
|  | 0.382792012 |  |
|  | 0.358869688 |  |
|  | 0.347856997 |  |
|  | 0.345670923 |  |
|  | 0.363425179 |  |
|  | 0.343637395 |  |
|  | 0.317970526 |  |
|  | 0.34302408 |  |
|  | 0.287809756 |  |
|  | 0.315498293 |  |
|  | 0.391168975 |  |
|  | 0.367608674 |  |
|  | 0.405422324 |  |
|  | 0.387223809 |  |
|  | 0.330903477 |  |
|  | 0.316190443 |  |
|  | 0.348290669 |  |
|  | 0.321837313 |  |
